# Building the Evidence Base for Oxford Surgical Innovation: An Effective Platform for Supporting Innovation in Surgery

**DOI:** 10.1177/1553350620942979

**Published:** 2020-07-09

**Authors:** Marta de Andres Crespo, Solveig Hoppe, James McVeigh, Matthew A. Williams, Ashok I. Handa

**Affiliations:** 1 Nuffield Department of Surgical Sciences, 6396 University of Oxford, UK

Dear Editor,

Following the release of the Royal College of Surgeons’ “Future of Surgery” report, we recently wrote a letter describing the launch of a novel platform for the promotion of safe and successful innovation in surgery: The Oxford Surgical Innovation Conference (OxSI).^1^ After collating feedback from our second event, OxSI 2020, we write to you now to further evidentiate OxSI as an effective platform for meeting predefined learning objectives.

OxSI 2020 was held at St Catherine’s College, Oxford, on March 13, 2020. The day consisted of five keynote lectures delivered through a plenary session, as well as a choice of five interactive breakout sessions that delegates self-selected prior to the event. Together, these learning modalities sought to inform delegates on our key learning objectives:

1. To educate delegates on the practicalities of engaging in safe and successful innovation in surgery;
2. To present delegates with examples of ongoing innovation in surgery to contextualise these processes.

After the close of the conference, feedback was collected from delegates by means of a four-point Likert-style questionnaire. This showed that delegates were highly satisfied with both the plenary session and the workshops, with average scores of 3.6 and 3.5 respectively. Pre- and post-conference delegate questionnaires demonstrated a statistically significant self-reported improvement in our key learning objectives: understanding of practicalities +1.15 (*P* < .0001) and ability to discuss current innovations +1.18 (*P* < .0001). In free-text comments, delegates praised the interactive and current nature of the course.

Feedback from OxSI 2020 has been wholly positive, and we are seeking to further develop the evidence behind this novel platform. We believe the results presented here support OxSI as an effective tool for promoting safe and successful innovation in surgery. Given these exciting results, we very much look forward to OxSI 2021.
